# Return-to-learn after concussion in Washington state public high schools during the COVID-19 pandemic

**DOI:** 10.2217/cnc-2022-0011

**Published:** 2023-02-13

**Authors:** Shyam J Deshpande, Aspen Avery, Julian Takagi-Stewart, Brianna Mills, Qian Qiu, Monica S Vavilala

**Affiliations:** ^1^Department of Anesthesiology & Pain Medicine, University of Washington, Seattle, WA 98195, USA; ^2^Department of Pediatrics, University of Washington, Seattle, WA 98195, USA; ^3^Harborview Injury Prevention & Research Center, Seattle, WA 98122, USA; ^4^Department of Epidemiology, University of Washington, Seattle, WA 98195, USA

**Keywords:** concussion, COVID-19, return-to-learn, traumatic brain injury

## Abstract

**Aim:** To understand academic support structures for Washington state public high school students with concussion during the COVID-19 pandemic. **Materials & methods:** Prospective, repeated cross-sectional study of 21 schools in 2020 and 2021. **Results:** About 28% of schools reported not providing any return-to-learn (RTL) accommodations for students with concussion throughout the COVID-19 pandemic. RTL accommodation provision was associated with larger student body size (*β* = 0.002) and higher graduation rate (*β* = 0.261) but was not associated with presence of RTL school policy. About 38.1% of schools received no guidance on how to provide RTL accommodations during the COVID-19 pandemic, and many reported that students with concussion struggled more. **Conclusion:** Schools struggled to provide RTL accommodations for students with concussion during the COVID-19 pandemic, highlighting the need for evidence-based guidance and resource allocation to vulnerable schools.

## Tweetable abstract

This prospective, repeated cross-sectional study demonstrates that schools are struggling to provide academic support for students with concussion during the COVID-19 pandemic, highlighting the need for evidence-based guidance and resource allocation to vulnerable schools.

Concussion among youth is a major public health concern in the USA [1]. Accounting for over 2 million outpatient visits and 3 million emergency department visits between 2005 and 2009 [1], concussion occurs in 3.6–7.0% of youth less than 18 years over childhood [1] and is especially common in athletes [2,3]. Adolescents 13–17 years are the highest risk pediatric age group, with a prevalence of 6.5–18.3% [4]. Concussion can cause physical, emotional and cognitive symptoms, including headache, fatigue, slowed mentation, difficulty concentrating and sensitivity to light and noise [5]. These symptoms can last days to months [5,6] and are more likely to be prolonged in females or children with history of anxiety, depression or migraine [7].

Concussion can negatively impact academic performance in schools [8]. Children who sustain concussion have higher rates of cognitive impairment compared with peers without concussion and may be more likely to have a lower grade point average [7], though a paucity of quantitative performance data exists [9]. To promote recovery and successful return to school, current return-to-learn (RTL) guidelines recommend academic accommodations for students who have sustained a concussion [10], which may include cognitive and physical rest for the first several days after injury, followed by graduated return to academic activities, individualized symptom-based learning plan and ongoing reassessment with formal assistance. The CDC recommends a team-based approach and coordinated support within schools, and emphasizes the importance of education for school and healthcare professionals [11]. The five components of an effective RTL program according to the CDC are: identification, screening and assessment practices; systematic communication between medical and educational systems; student tracking over time; professional development for school personnel; outcome measures to assess academic success [12].

In Washington state, the University of Washington and Harborview Injury Prevention & Research Center have examined standardized RTL programs to better support students after concussion [13,14]. Ongoing efforts include the state-wide Return-to-Learn Implementation Bundle for Schools (RISE) After Youth Concussion trial [14]. The RISE trial is championed by local school staff representatives (RTL champions) and assesses the effects of an RTL implementation bundle on schools RTL protocols as well as student academic outcomes after concussion [13].

The SARS-CoV-2 virus was first reported in December 2019 [15], and the first case of COVID-19 in the USA occurred in Washington state in January 2020 [16]. By mid-March 2020 the Governor of Washington state ordered that schools close until late April 2020 [17,18]. The COVID-19 pandemic interrupted ongoing RTL efforts and forced schools to adjust curricula structure, primarily to virtual learning platforms [19]. These changes resulted in increased screen-time, which is associated with prolonged post-concussive symptoms [20] and likely altered communication and reporting pathways among school staff, students and families, all without the typical in-person support systems. Unmet RTL needs for students with concussion have been documented in Washington state prior to the pandemic (e.g., lack of school RTL policies, ongoing barriers to RTL accommodations and need for guidance on RTL implementation) [21], but how the pandemic affected academic experience for students with concussion is unknown.

The aim of this study was to understand RTL accommodations, school RTL policies and perception of student struggles for Washington state high school students with concussion during the COVID-19 pandemic and in the alternative learning environments.

## Materials & methods

### Participants

We conducted a prospective, repeated cross-sectional study of the Washington state public high school RTL champions enrolled in the RISE trial [14]. Schools were initially recruited to participated in the RISE trial via a state-wide iterative email recruitment process. A total of 460 schools were contacted by the study coordinator; 47 schools responded with interest. After excluding private schools, duplicates, schools not desiring concussion support and schools who declined participation after learning details of the RCT, 26 schools were eligible for enrollment, and a total of 21 schools completed enrollment by March 2021. RTL champions were school staff members (teachers, nurses, athletic trainers or principals) who participated as the point of contact for this work. All schools enrolled in the RISE clinical trial at the time of each survey were invited to participate.

### Instrumentation/procedure

RTL champions were sent electronic surveys in March 2020, April 2020 and March 2021. Both 2020 surveys included open-ended short-response questions that inquired about learning platform (i.e., no school, fully online, hybrid or in-person) and provisions of RTL accommodations for students with concussion (Supplementary Figure 1). Based on feedback for survey ease of completion, the 2021 survey questions used standardized checkbox questions to inquire about learning platform, types and number of RTL accommodations provided, types and number of RTL school policies in place, if schools received guidance on providing RTL accommodations during the pandemic and whether RTL champions felt students with concussion were struggling more with academics during the pandemic compared with prepandemic period. The 2021 survey also included a free-response section for additional comments (Supplementary Figure 2). The University of Washington Institutional Review Board deemed this study exempt from review.

Student demographics and school characteristics for the 2020 – 2021 academic year were abstracted from the Washington Office of Superintendent of Public Instruction (OSPI) and Washington Education Data and Research Center (EDRC) [22.^23^].

### Data analysis

Student demographics and school characteristics were compared using descriptive statistics. We examined learning platform type, RTL accommodations (presence, number and type), RTL policies (presence, number and type), sources of RTL guidance for schools, and perception of whether students with concussions struggled more during the COVID-19 pandemic in March 2020, April 2020 and March 2021.

Of the schools that completed the 2021 survey and at least one survey in 2020, learning platform was compared between schools at each survey time point.

For schools completing the survey in March 2021, we examined the association between RTL accommodations provided with school characteristics, as well as the association between RTL accommodations provided and presence of RTL school policy, using linear regression and ANOVA (outcome variable was the number of RTL accommodations provided, or whether a school provided any vs no accommodations). We then compared source of RTL guidance with school factors, as well as the perception of student struggles with school factors, using descriptive statistics. Qualitative comments written in the 2021 survey were evaluated for common themes.

Study data were collected and managed using REDCap electronic data capture tools hosted at the University of Washington [24]. The STROBE cross sectional reporting guidelines were used [25]. Data were processed using R Version 3.6.1 [26].

## Results

### Characteristics

A total of 21 schools were enrolled in the RISE trial. Sixteen of 21 schools had enrolled in the RISE trial by 2020 and completed at least one survey in 2020 plus the survey in 2021 (four of 16 responded only in March 2020 and March 2021, three of 16 responded only in April 2020 and March 2021 and nine of 16 schools responded to all three surveys). Five of 21 schools were enrolled after the surveys were issued in 2020, and, therefore completed the survey in March 2021 only. Student body demographics and school characteristics of the 21 schools that completed the March 2021 survey are shown in Table 1. As reported by OSPI and EDRC, during the 2020–20201 academic year, the student bodies of participating schools were approximately 49% female, 58% white and 27% Hispanic/Latino. An average of 41% of students received free or reduced lunch, approximately 6% of students had a 504 plan and approximately 13% of students had a disability. Most (81%) schools were urban. Schools had on average 1433 students, a 91.6% 4-year graduation rate, 17.7 students per class and 11.4 students to one teacher. During the 2020–2021 academic year, participating schools spent an average of US$13,821 per student.

**Table 1.** Characteristics of 21 participating Washington state public high schools during the 2020–2021 academic year and COVID-19 pandemic.

Characteristic	Value (n = 21)
Student gender	
Male (%)	51.23 (1.73)
Female (%)	48.67 (1.71)
Gender X (%)	0.11 (0.15)

Student race/ethnicity	
White (%)	57.72 (26.30)
Hispanic/Latino of any race(s) (%)	26.67 (26.46)
Two or more races (%)	6.61 (4.38)
Asian (%)	4.48 (7.02)
Black or African–American (%)	1.95 (3.33)
American–Indian/Alaska Native (%)	1.90 (4.03)
Native Hawaiian/Other Pacific Islander (%)	0.64 (1.55)

School feature	
Rural (n)	4 (19.0)
Urban (n)	17 (81.0)
Student body size (n)	1142.52 (570.60)
Expenditure per student per year (US$)	13821.90 (1633.28)
Graduation rate in 4 years (%)	91.62 (5.00)
Mean class size (n)	17.67 (8.63)
Student-to-teacher ratio (n)	11.43 (3.59)

Equity	
Students receiving free/reduced lunch (%)	40.74 (25.08)
Students with disabilities (%)	12.60 (3.14)
Students with a 504 plan (%)	5.67 (4.74)

Continuous variables listed as mean (standard deviation). Categorical variables listed as n (%).

### Learning platforms

Of the 16 schools that completed the 2021 survey and at least one survey in 2020, 53.8% (7/13 schools) had no school in March 2020, 91.7% (11/12 schools) were fully online in April 2020 and 93.8% (15/16 schools) had hybrid curricula in March 2021 (Supplementary Table 1).

### RTL accommodations

The number of schools providing RTL accommodations changed over the study period. Of the 16 schools that responded to the 2021 survey and at least one survey in 2020, the percentage of schools that provided any RTL accommodation was 30.8% (4/13 schools) in March 2020, 83.3% (10/12 schools) in April 2020 and 75% (12/16 schools) in March 2021.

**Figure 1.**
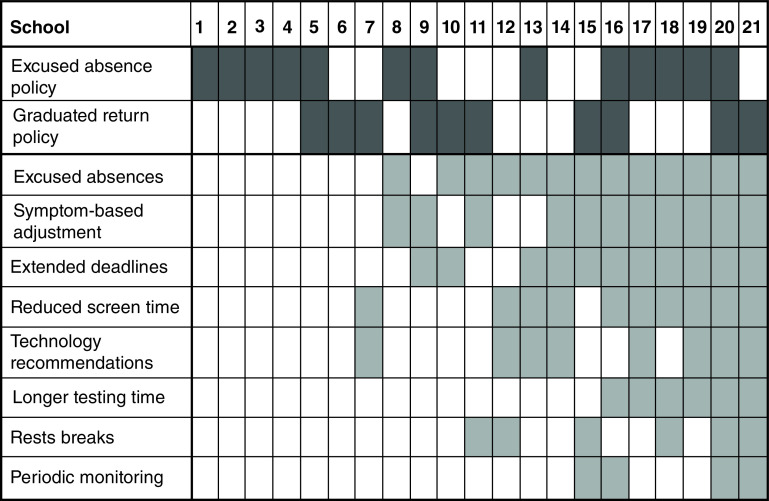
Categories of return-to-learn accommodations provided and return-to-learn policy in place for students with concussion by 21 participating Washington state public high schools in March 2021. Shaded boxes = accommodation offered/ policy in place.

The type and number of RTL accommodations provided by the 21 participating schools in March 2021 is shown in Figure 1. Fifteen (71.4%) of the 21 schools provided RTL accommodations, whereas six (28.6%) schools provided no RTL accommodations. For schools providing accommodations, the average number and standard deviation of RTL accommodations provided was 4.6 ± 2.1. The most common types of accommodations provided were excused absences (13/21 schools), symptom-based adjustment (11/21 schools), extended deadlines (11/21 schools) and reduced screen time (10/21 schools).

Table 2 shows the association between number of unique RTL accommodations provided with school characteristics and student body demographics. There were statistically significant associations between number of accommodations provided and student body size (*β* = 0.002; 95% CI: 0.000, 0.004) and with 4-year graduation rate (*β* = 0.261; 95% CI: 0.028–0.495), meaning for every increase of 500 students or 3.8% in graduation rate, a school would provide one additional type of accommodation. There were no statistically significant associations between the number of accommodations provided and the percentage of the student body that was female, was white, received free/reduced lunch, had a 504 plan or had a disability. Nor were there statistically significant associations between the number of accommodations provided and urban versus rural school designation, learning platform, annual expenditure per student or mean class size.

**Table 2.** Association between number of types of return-to-learn accommodations provided with characteristics of 21 participating Washington state public high schools during the 2020–2021 academic year.

Characteristic	Linear regression *β*-coefficient (95% CI)
Rural vs urban	‐0.265 (-3.555–3.026)
Curriculum:	
– Fully in-person vs hybrid	5.000 (-2.028–12.028)
– Fully in-person vs full online	2.389 (-1.888–6.666)
Student body (n)	0.002 (0.000–0.004)^†^
Expenditure per student per year (US$)	‐0.000 (-0.001–0.000)
Rate of graduation in 4 years (%)	0.261 (0.028–0.495)^†^
Mean class size (n)	0.031 (-0.122–0.184)
Female (%)	‐0.208 (-0.974–0.559)
White (%)	‐0.005 (-0.046–0.055)
Students receiving free/reduced lunch (%)	‐0.031 (-0.082–0.020)
Students with 504 plan (%)	0.230 (-0.027–0.486)
Students with disabilities (%)	‐0.342 (-0.731–0.047)

† Statistically significant.

### RTL policy

School RTL policy options for students with concussion on the March 2021 survey included excused absence policies (“Enable absence from school during recovery from concussion, and then return to a full load of academic tasks based on a note from parent or healthcare provider”), graduated return policies (“Provide regular assessments and corresponding accommodations based on student reported symptoms directly to schools”) or none. Almost all of the schools (19/21 schools) had an RTL policy in place in March 2021 (Supplementary Table 2). Of the 21 schools, nine (42.9%) had excused absence policies, six (28.6%) had graduated return policies, four (19.0%) had both absence and graduated return policies and two (9.5%) had none. There were no statistically significant differences in student demographics or school characteristics between schools with the various policies. There was no statistically significant association between whether a school offered no accommodations versus any accommodations with the number of RTL policies in place (OR: 0.696; 95% CI: 0.101, 4.409), nor was there an association between the number of RTL accommodations offered with any specific policy type (F = 0.33, p = 0.802).

### RTL guidance

**Figure 2.**
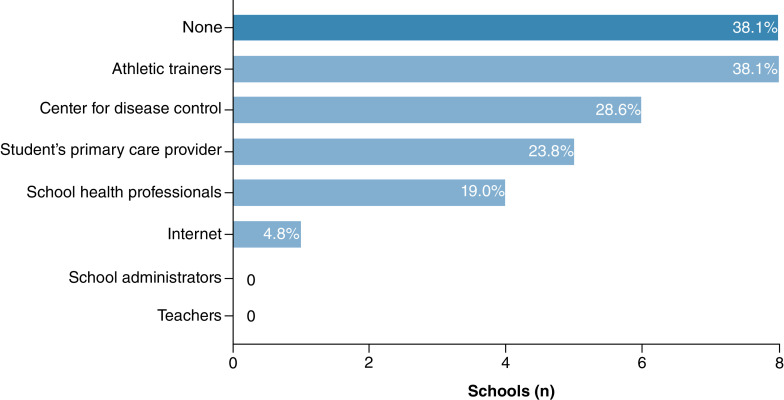
Source of guidance on return-to-learn accommodations for students with concussion from 21 participating Washington state public high schools in March 2021.

Figure 2 illustrates where schools received guidance regarding RTL accommodations during the COVID-19 pandemic. Approximately 38% (8/21 schools) of schools reported that they did not receive any guidance on how to provide RTL accommodations during the COVID-19 pandemic. The most common sources of guidance were athletic trainers (8/21 schools) followed by the CDC (6/21 schools). No schools received guidance from school administrators.

### COVID-19 pandemic struggles

RTL champions in March 2021 responded to the question: “Do you think that compared with prepandemic time, students with concussion are struggling with academics more during the pandemic?” Approximately half (10/21 schools) reported that students with concussion were struggling more, and almost all the other RTL champions (10/21 schools) were uncertain (Supplementary Table 3). Only one school felt that students with concussion were not struggling more. There were no statistically significant differences in the number of RTL accommodations provided, the number of RTL policies in place, student body demographics or school characteristics between schools that reported an increase in student struggles versus those that did not.

### RTL themes

Thirteen schools submitted free text comments in the March 2021 survey (Supplementary Table 4). The most common theme described was a decrease in concussion incidence during the pandemic due to reduction in organized school sports. Additionally, RTL champions frequently expressed that increased screen time during the pandemic negatively impacted students with concussion, and RTL champions reported that increased screen time prolonged concussion recovery. RTL champions also described that symptom follow-up for students with concussion was more difficult during remote learning, but some were able to accomplish follow-up through online video platforms or electronic correspondence.

## Discussion

The overall purpose of this study was to understand the effect of the COVID-19 pandemic on students with concussion, and we had such an opportunity to do so in Washington state. After closure of Washington state schools in March 2020 [17,18], schools acclimatized learning platform, transitioning from no school, to fully in-online, to hybrid curricula 1 year later. Our study shows that: over 1/4 of schools did not provide any type of RTL accommodations for students with concussion; there was considerable inconsistency between Washington state public high schools in RTL structure; many schools struggled to provide RTL accommodations even if they had RTL policies in place; over 1/3 of schools received no guidance on how to provide RTL accommodations during the pandemic; and larger schools with more resources that support graduation may have had more capacity to provide RTL accommodations. Together, these findings suggest that the COVID-19 pandemic posed significant symptom recovery and RTL accommodation challenges for Washington state public high school students with concussion. To our knowledge, this is the first study to define the unique difficulties and barriers faced by public high school students with concussion during the COVID-19 pandemic.

In this study, we report a gap between RTL policy and accommodation practice. This disparity may be due to educational factors (lack of quality policy education materials, or failure to provide protected time for education), cost of implementation (financial costs and staff work required) [27], misunderstanding of policy versus recommendation [28], stakeholders’ personal impressions of the policy’s value or failure to provide guidance (via reliance on teachers without providing examples of implementation) [29].

Concerningly, many schools also lacked RTL guidance early during the pandemic on how to provide RTL accommodations and principally relied on the expertise of athletic trainers. However, only 61% of Washington state public high schools regularly have access to athletic trainers and how their role in detection and treatment of concussion symptoms changed during the pandemic is not clear [30]. Furthermore, public schools in Washington state and nationally that are rural, smaller in student body, have a higher proportion of students receiving free/reduced lunch and have higher proportion of students with lower median household income are even less likely to have access to athletic trainers [31,32]. This means that not only were schools not receiving the resources they needed in order to support students during this alternative learning environment, but that vulnerable schools were likely disproportionately disadvantaged, widening disparities during the pandemic. These findings suggest that students from less well-resourced schools may be further left behind academically by the time schools return to in-person learning platforms. Furthermore, increased attention may need to be paid to students with concussion who may not have received the care they needed at the peak of the pandemic. These findings represent an opportunity for unified guidance from school boards and policymakers, as well as resource allocation to vulnerable schools, including athletic training support, school health concussion follow-up, RTL educational material and protected time for stakeholders to devise and implement RTL accommodations in the classroom.

Schools subjectively reported fewer students with concussion during the COVID-19 pandemic than before the pandemic. These observations are supported in published literature [33]. Kontos and colleagues reported that there were overall and proportionally fewer pediatric and sport-related concussions evaluated at a concussion clinic in Pennsylvania during the COVID-19 pandemic. This is not surprising given the overall burden of organized sport-related concussion as a principal cause in children [34]. However, this study reveals that students with concussion across demographics struggled more during the COVID-19 pandemic, despite RTL accommodations or existing RTL policies. We think that this may be due to a combination of increased reliance on screen time, lack of typical in-person support structures and the mental health impact of the pandemic and social isolation [35], on top of existing concussion-related emotional and cognitive symptoms.

Limitations of this study include convenience sample methodology and sample size, which may reduce generalizability of results and lack of baseline prepandemic data and variation in survey instrument, which had to be adapted due to the given constraints of the COVID-19 pandemic. These data rely on the knowledge and insight of designated RTL champions in each school and are subject to recall and observation bias. Whereas, survey questions on RTL accommodations intended to elicit the accommodations being provided at a given time, it is possible that respondents may have answered based on accommodations they understand a school would typically provide. We could not examine how specific types of RTL accommodations or the perception of student struggles changed over the study period.

## Conclusion

Our work is the first to describe RTL accommodations, school RTL policy and perception of student experience after concussion during the COVID-19 pandemic. It is clear that public high schools are struggling to provide RTL accommodations, regardless of student demographics, schools characteristics or RTL policies and that small and less well-resourced schools appear to struggle more with providing RTL accommodations. Schools lack guidance on how to provide accommodations during this alternative learning environment, and students with concussion are struggling even more with concussion recovery and school academics than during the prepandemic period. This study highlights an opportunity for policy makers, educators and healthcare professionals to provide schools with evidence-based RTL guidance as well as to allocate resources to vulnerable schools to better support students with concussion. Future research should examine RTL accommodations, concussion symptom follow-up and school experience as schools transition back to in-person learning.

**Summary points**

- The COVID-19 pandemic disrupted normal academic support systems for students with concussion in Washington state public high schools.
- Schools struggled to provide return-to-learn (RTL) accommodations during the COVID-19 pandemic.
- Over 1/4 of schools did not provide any type of RTL accommodations for students with concussion from March 2020 through March 2021.
- Many schools struggled to provide RTL accommodations even if they had formal RTL policies in place.
- Over 1/3 of schools received no guidance on how to provide RTL accommodations during the COVID-19 pandemic.
- Students with concussion at smaller and less well resourced schools may have been disproportionately impacted by the COVID-19 pandemic.
- Students with concussion likely struggled academically more during the COVID-19 pandemic than before.
- This study highlights an opportunity for policy makers, educators and healthcare professionals to provide schools with evidence-based RTL guidance as well as to allocate resources to vulnerable schools.
