# Supplementary material for: Return-to-learn after concussion in Washington state public high schools during the COVID-19 pandemic
Source: Concussion. 2023 Feb 13;8(2):CNC103. doi: 10.2217/cnc-2022-0011 (PMC9937029; doi:10.2217/cnc-2022-0011)
Supplement: Supplementary file 1 [file cnc-08-103-s1.pdf]

Supplemental Figure 1. Electronic survey sent to Return-to-Learn champions in Washington state public high schools in March 2020 and/or April 2020.

---

1. What school do you work for?

____

---

2. Please provide your name and title.

____

---

3. During Washington state school closure, have your school migrated or are migrating to online learning ?

◯ Yes
◯ No

3a. Please describe your online program
(e.g. formal online program with attendance and assignments; teachers posting ideas for learning that have no requirements).

____

---

4. How many hours per day is your online educational curriculum ?

____

4a. How did you decide on the number of hours ?

____

4b. Do you provide accommodations for those who cannot tolerate online education (please describe if any)?

____

4c. Do you have any current students diagnosed with a concussion that would be required to complete this online education platform?

____

4d. What accommodations can you provide for those with concussion whose symptoms get worse with screen time ?

____

4e. How do you decide when students can resume online learning ?

____
