# Supplementary material for: Return-to-learn after concussion in Washington state public high schools during the COVID-19 pandemic
Source: Concussion. 2023 Feb 13;8(2):CNC103. doi: 10.2217/cnc-2022-0011 (PMC9937029; doi:10.2217/cnc-2022-0011)
Supplement: Supplementary file 2 [file cnc-08-103-s2.pdf]

Supplemental Figure 2. Electronic survey sent to Return-to-Learn champions in Washington state public high schools in March 2021.

---

1. Your name:

____

---

2. Your school:

____

---

3. Do you still work for the school that you served as an RTL champion for in 2020?

◯ Yes
◯ No

---

4. Are you still acting as the RTL champion?

◯ Yes
◯ No

4a. Can you tell us if a replacement was made at your school?

◯ Yes
◯ No
◯ N/A

5. What is your current school format?

◯ Fully online
◯ Fully in-person
◯ Hybrid (combination of online and in-person)

---

6. What academic accommodations did the teachers at your school provide for students with a concussion during the pandemic? (Select all that apply)

☐ Rests breaks between classes
☐ Excused absences from classes or school
☐ Technology recommendations
☐ Reduced screen time
☐ Symptom based adjustments
☐ Longer testing time
☐ Extended deadlines for homework or projects
☐ Periodic monitoring of concussion symptoms
☐ None
☐ Did not have concussed students needing academic accommodations

---

7. Has your school sent any communications to parents about academic accommodations for concussion during the pandemic? (Select all that apply)

☐ Was sent to all parents
☐ Was sent only to parents of student with concussion
☐ Was sent by email
☐ Was mailed to home address
☐ Included a contact person
☐ Was sent more than once

---

8. Where did you receive your guidance regarding academic accommodations for concussions that would be needed to support students during the pandemic? (select all that apply)

☐ School health professional
☐ CDC Guidance
☐ Internet
☐ Student's Primary Care Provider
☐ School administrator
☐ Teachers
☐ Athletic Trainers
☐ I have not received any guidance regarding academic accommodations for concussions during the pandemic

---

9. During the pandemic, how often did students with concussion request academic accommodations for a concussion?

◯ Never
◯ Rarely
◯ Sometimes
◯ Often
◯ Always

---

10. What is your school's current guideline on accommodating students with concussions during the pandemic? (select all that apply)

☐ Enable absence from school during recovery, and then return to a full load of academic tasks based on a note from parent or health care provider
☐ Provide regular assessments and corresponding accommodations based on student reported symptoms directly to schools
☐ No accommodations are given for concussions during the pandemic

11. Do you think that compared to pre-pandemic time, students with concussion are struggling with academics more during the pandemic?

◯ Yes
◯ No
◯ Uncertain

12. Would you like to tell us anything else about academic accommodations for concussion during the pandemic?
