# Supplementary material for: Return-to-learn after concussion in Washington state public high schools during the COVID-19 pandemic
Source: Concussion. 2023 Feb 13;8(2):CNC103. doi: 10.2217/cnc-2022-0011 (PMC9937029; doi:10.2217/cnc-2022-0011)
Supplement: Supplementary file 3 [file cnc-08-103-s3.docx]

**Supplemental Table 1.** Learning platform of 16 participating Washington state public high schools that completed at least one survey in 2020 and the 2021 survey. Variables listed as number (%).

| **Learning platform** | **March 2020**  N=13 | **April 2020**  N=12 | **March 2021**  N=16 |
| --- | --- | --- | --- |
| Fully in-person | 0 | 0 | 0 |
| No school | **7 (53.8)** | 1 (8.3) | 0 |
| Fully online | 6 (46.2) | **11 (91.7)** | 1 (6.2) |
| Hybrid online | 0 | 0 | **15 (93.8)** |
