# Supplementary material for: Return-to-learn after concussion in Washington state public high schools during the COVID-19 pandemic
Source: Concussion. 2023 Feb 13;8(2):CNC103. doi: 10.2217/cnc-2022-0011 (PMC9937029; doi:10.2217/cnc-2022-0011)
Supplement: Supplementary file 4 [file cnc-08-103-s4.docx]

**Supplemental Table 2.** Return to Learn (RTL) policies in place in March 2021 of 21 participating Washington state public high schools. Continuous variables listed as mean (SD). Categorical variables listed as number (%).

| **Characteristic** | **RTL policy type** | | | |
| --- | --- | --- | --- | --- |
|  | **None**  **(n=2)** | **Excused absence**  **(n=9)** | **Graduated return**  **(n=6)** | **Both**  **(n=4)** |
| **Student gender** | | | | |
| Male (%) | 50.55 (2.47) | 51.23 (1.86) | 50.77 (1.93) | 52.25 (0.54) |
| Female (%) | 49.25 (2.19) | 48.73 (1.84) | 49.08 (1.99) | 47.60 (0.52) |
| Gender X (%) | 0.25 (0.21) | 0.04 (0.05) | 0.13 (0.15) | 0.15 (0.24) |
| **Student race / ethnicity** | | | | |
| White (%) | 64.65 (1.06) | 60.01 (25.36) | 50.15 (37.77) | 60.48 (18.61) |
| Hispanic/ Latino of any race(s) (%) | 27.80 (3.82) | 23.24 (27.94) | 36.02 (35.75) | 19.80 (13.40) |
| Two or more races (%) | 4.20 (0.85) | 6.56 (3.90) | 5.87 (4.62) | 9.05 (6.20) |
| Asian (%) | 1.65 (1.34) | 7.02 (10.01) | 1.65 (1.13) | 4.40 (4.17) |
| Black or African American (%) | 0.80 (0.14) | 2.18 (2.83) | 0.63 (0.98) | 4.00 (6.38) |
| American Indian/ Alaska Native (%) | 0.75 (0.21) | 0.59 (0.43) | 5.22 (6.77) | 0.42 (0.53) |
| Native Hawaiian/ Other Pacific Islander (%) | 0.10 (0.00) | 0.34 (0.32) | 0.43 (0.92) | 1.88 (3.42) |
| **School feature** | | | | |
| Rural (n) | 0 (0.0) | 1 (11.1) | 2 (33.3) | 1 (25.0) |
| Urban (n) | 2 (100.0) | 8 (88.9) | 4 (66.7) | 3 (75.0) |
| Student body size (n) | 1332.50 (378.30) | 1217.11 (596.61) | 881.00 (487.91) | 1272.00 (759.60) |
| Expenditure per student per year ($) | 13042.00 (1530.18) | 13096.11 (1407.00) | 15076.83 (786.57) | 13962.50 (2351.91) |
| Graduation rate in 4 years (%) | 90.45 (0.49) | 92.39 (4.21) | 93.42 (3.61) | 87.78 (8.30) |
| Mean class size (n) | 17.50 (0.71) | 17.00 (2.92) | 19.83 (16.27) | 16.00 (4.32) |
| Student-to-teacher ratio (n) | 15.00 (7.07) | 11.11 (3.33) | 10.00 (0.00) | 12.50 (5.00) |
| **Equity** | | | | |
| Students receiving free/ reduced lunch (%) | 39.85 (12.09) | 33.04 (25.93) | 55.32 (25.14) | 36.62 (25.94) |
| Students with disabilities (%) | 13.75 (0.92) | 11.83 (3.08) | 14.58 (3.04) | 10.80 (3.11) |
| Students with 504 plan (%) | 5.20 (1.98) | 7.06 (6.66) | 4.07 (2.39) | 5.17 (3.06) |
