# Supplementary material for: Return-to-learn after concussion in Washington state public high schools during the COVID-19 pandemic
Source: Concussion. 2023 Feb 13;8(2):CNC103. doi: 10.2217/cnc-2022-0011 (PMC9937029; doi:10.2217/cnc-2022-0011)
Supplement: Supplementary file 5 [file cnc-08-103-s5.docx]

**Supplemental Table 3.** Association of Return to Learn (RTL) champion perception that students with concussion are struggling more with academics during the COVID-19 pandemic (in comparison to pre-pandemic) with characteristics of 21 participating WA public high schools during the 2020 - 2021 academic year.

| **School Characteristic** | **Perception of Student Struggles More During the Pandemic** | |
| --- | --- | --- |
|  | **Uncertain (n=10) / No (n=1)** | **Yes (n=10)** |
| Number of unique RTL accommodations (n) | 3.36 (2.84) | 3.20 (2.82) |
| Number of unique RTL policies (n) | 1.27 (0.65) | 0.90 (0.32) |
| **Student gender** | | |
| Male (%) | 51.14 (1.48) | 51.33 (2.04) |
| Female (%) | 48.72 (1.41) | 48.61 (2.08) |
| Gender X (%) | 0.14 (0.17) | 0.08 (0.12) |
| **Student Race** | | |
| White (%) | 56.10 (28.86) | 59.51 (24.61) |
| Hispanic/ Latino of any race(s) (%) | 30.43 (28.99) | 22.54 (24.22) |
| Two or more races (%) | 6.23 (4.81) | 7.03 (4.06) |
| Asian (%) | 2.64 (2.87) | 6.50 (9.57) |
| American Indian/ Alaska Native (%) | 2.13 (4.98) | 1.64 (2.89) |
| Black or African American (%) | 1.70 (3.95) | 2.23 (2.66) |
| Native Hawaiian/ Other Pacific Islander (%) | 0.78 (2.07) | 0.48 (0.71) |
| **School feature** | | |
| Rural (n) | 3 (27.3) | 1 (10.0) |
| Urban (n) | 8 (72.7) | 9 (90.0) |
| Student body size (n) | 1128.91 (496.14) | 1157.50 (670.47) |
| Expenditure per student per year ($) | 13782.45 (1757.87) | 13865.30 (1578.16) |
| Graduation rate in 4 years (%) | 89.76 (5.95) | 93.66 (2.71) |
| Mean class size (n) | 15.55 (3.78) | 20.00 (11.76) |
| Student-to-teacher ratio (n) | 12.73 (4.67) | 10.00 (0.00) |
| **Equity** | | |
| Students receiving free/ reduced lunch (%) | 42.53 (26.89) | 38.77 (24.21) |
| Students with disabilities (%) | 12.78 (3.79) | 12.41 (2.42) |
| Students with 504 plan (%) | 6.08 (5.60) | 5.21 (3.83) |
