# Supplementary material for: Return-to-learn after concussion in Washington state public high schools during the COVID-19 pandemic
Source: Concussion. 2023 Feb 13;8(2):CNC103. doi: 10.2217/cnc-2022-0011 (PMC9937029; doi:10.2217/cnc-2022-0011)
Supplement: Supplementary file 6 [file cnc-08-103-s6.docx]

**Supplemental Table 4.** Themes of qualitative comments submitted from 13 participating Washington state public high schools in March 2021.

| **Theme** | **Quotes** |
| --- | --- |
| Decline in sports-related concussions (n=6) | 1. “Just started up sports on 2/22 and have not had any record of sustained concussions at this point” 2. “We have had significantly fewer concussions this year.” 3. “In the middle of our amended athletic seasons” 4. “We returned to sports approx. 2/8/21. Prior to that I only knew of one student with a concussion from a motor vehicle crash” 5. “Concussion rate went down significantly this year because we didn't do any sports.” 6. “I have double checked with Athletic department and attendance secretary, no new concussions have been diagnosed this school year” |
| Challenges of increased screen time (n=5) | 1. “Our district did not have paper alternative to screens which again was hard for concussed students.” 2. “Screen time would have been our concern as we just now returned to hybrid in-person.” 3. “Much more difficulty with work load and accommodations for students with symptomatic concussion - due all learning being remote on screen…Recovery seems to be longer than when students were in person.” 4. “I think the increased screen time required for hybrid school is causing increased concussion symptoms in our students that require more breaks and possible longer recovery time.” 5. “Reduction of screen time means reduction at all remote learning” |
| Difficulty of remote concussion follow-up (n=4) | 1. “My district told me I was not to contact students directly on the phone. It made follow up difficult.” 2. “For those students who had concussions… I scheduled Zoom meetings for symptom checks and would communicate with teachers after each meeting.” 3. “I haven't been in touch with any students over the pandemic concerning a concussion…lack of the normal access to myself as an athletic trainer.” 4. “I have been serving students remotely” |
| Description of school concussion protocol (n=1) | 1. “I have been following the return to learning protocol.” |
| Description of remote curriculum (n=1) | 1. “Our remote learning consisted of very limited class times (30 min) and options to view recorded class lectures.” |
